# 1201. Diphtheria in Veterans Health Administration (VHA), 2000-2021

**DOI:** 10.1093/ofid/ofab466.1393

**Published:** 2021-12-04

**Authors:** Patricia Schirmer, Cynthia A Lucero-Obusan, Aditya Sharma, Gina Oda, Mark Holodniy

**Affiliations:** Department of Veterans Affairs, Palo Alto, California

## Abstract

**Background:**

Diphtheria is caused by *Corynebacterium diphtheriae* and can cause respiratory or skin infections. Transmission occurs primarily person-to-person via respiratory tract and rarely from skin lesions or fomites. In the Veterans Health Administration (VHA), we perform surveillance for nationally notifiable diseases such as diphtheria. In early 2021, there were 4 alerts for *C. diphtheriae*. Therefore, we investigated diphtheria prevalence in VHA over the last 20 years.

**Methods:**

Isolates of *C. diphtheriae* were identified from VHA data sources from 1/1/2000-2/28/2021. Patient demographics, co-morbidities, microbiologic data, treatment, outcomes, and vaccination status were obtained via electronic medical record (EMR) review.

**Results:**

33 *C. diphtheriae* isolates were identified representing 32 unique individuals. 17 isolates were identified from 2000-2015 and 16 were identified from 2016-2021. Isolates were from cutaneous (16), blood (10), urine (4), pulmonary (2), and throat (1) specimens. In 11 individuals, clinical significance was unclear (no antibiotics given, note mentioned that it was being considered a contaminant - i.e., isolate may have been incorrectly labeled as “*C. diphtheriae*” instead of “diphtheroid”). Only 3 isolates had toxin testing documented. One *C. diphtheriae* biovar gravis blood isolate was associated with sepsis without another source identified. The throat isolate was a nontoxigenic strain. No cutaneous isolates underwent susceptibility testing, but all 15 individuals received antibiotics (1 patient had 2 isolates). 11 had additional organisms identified in addition to *C. diphtheriae*. Table 1 describes demographics, co-morbidities, and vaccination status of cutaneous cases. Only 1 case (in 2021) had EMR documentation of local public health department reporting.

Table 1. Characteristics of Unique Individuals with Cutaneous Diphtheria Isolates in VHA, 2000-2021

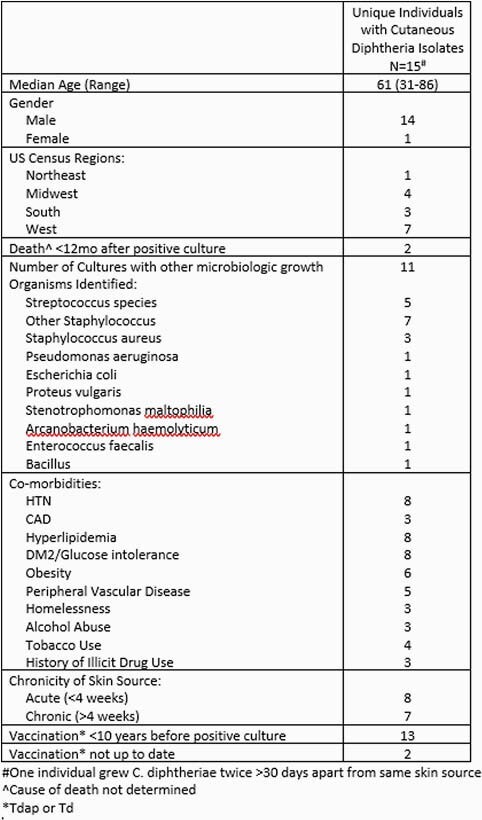

**Conclusion:**

Nearly as many isolates have been identified in the last 5.5 years compared to the previous 15 years which may be related to more robust molecular identification methods available in VHA. Most *C. diphtheriae* isolated was from cutaneous sources that were acute in onset. About 33% were identified as *C. diphtheriae* but were not treated. EMR documentation of toxin production and public health department reporting was lacking.

**Disclosures:**

**All Authors**: No reported disclosures

